# Comparing behavioural outcomes in children born extremely preterm between 2006 and 1995: the EPICure studies

**DOI:** 10.1007/s00787-023-02258-w

**Published:** 2023-07-11

**Authors:** Jennifer Larsen, Puja Kochhar, Dieter Wolke, Elizabeth S. Draper, Neil Marlow, Samantha Johnson

**Affiliations:** 1 Department of Population Health Sciences, George Davies Centre, University of Leicester, University Road, Leicester, LE1 7RH UK; 2 Institute of Mental Health, University of Nottingham, Nottingham, UK; 3 Neurodevelopmental Specialist Service (NeSS), Nottinghamshire Healthcare NHS Foundation Trust, Highbury Hospital, Nottingham, UK; 4 Department of Psychology and Warwick Medical School, University of Warwick, Coventry, UK; 5 EGA Institute for Women’s Health, University College London, London, UK

**Keywords:** Extremely preterm, Problem behaviour, Emotional regulation, Attention-deficit hyperactivity disorder, Social problems, Autism spectrum disorder

## Abstract

**Background:**

Children born extremely preterm (EP) are at increased risk of neurocognitive and behavioural morbidity. Here, we investigate whether behavioural outcomes have changed over time concomitant with increasing survival following EP birth.

**Methods:**

Comparison of outcomes at 11 years of age for two prospective national cohorts of children born EP in 1995 (EPICure) and 2006 (EPICure2), assessed alongside term-born children. Behavioural outcomes were assessed using the parent-completed Strengths and Difficulties Questionnaire (SDQ), DuPaul Attention-Deficit/Hyperactivity Disorder Rating Scale (ADHD-RS), and Social Communication Questionnaire (SCQ).

**Results:**

In EPICure, 176 EP and 153 term-born children were assessed (mean age: 10.9 years); in EPICure2, 112 EP and 143 term-born children were assessed (mean age: 11.8 years). In both cohorts, EP children had higher mean scores and more clinically significant difficulties than term-born children on almost all measures. Comparing outcomes for EP children in the two cohorts, there were no significant differences in mean scores or in the proportion of children with clinically significant difficulties after adjustment for confounders. Using term-born children as reference, EP children in EPICure2 had significantly higher SDQ total difficulties and ADHD-RS hyperactivity impulsivity z-scores than EP children in EPICure.

**Conclusions:**

Behavioural outcomes have not improved for EP children born in 2006 compared with those born in 1995. Relative to term-born peers, EP children born in 2006 had worse outcomes than those born in 1995. There is an ongoing need for long-term clinical follow-up and psychological support for children born EP.

**Supplementary Information:**

The online version contains supplementary material available at 10.1007/s00787-023-02258-w.

## Introduction

Compared with children born at term (≥ 37 week gestation), children born extremely preterm (EP; < 28 week gestation) are at increased risk of neurodevelopmental disabilities [1] and cognitive impairment [2, 3]. EP birth has also been shown to be a risk factor for behavioural problems, in particular inattention, internalising problems, and social-emotional difficulties [4–7].

Advances in neonatal care over the past 3 decades have resulted in improved survival following EP birth [8–12]. However, improvements in survival have not yet translated into improved long-term outcomes. Results from consecutive cohort studies found 4–17% more EP children survived to 1–3 years of age without neurodevelopmental impairment [13–15], despite no change in the rates of severe impairment [13, 15]. However, these improvements were not sustained at school age [16, 17]. There was no change in the prevalence of neurodevelopmental disability, cognitive impairment, and academic attainment between EP children born at 22–25 week gestation in England in 2006 and in 1995, in the EPICure and EPICure2 cohorts, respectively, at 11 years of age [17]. The Victorian Infant Collaborative Study (VICS) group also observed higher rates of motor impairment and poorer executive function and academic achievement at age 7–8 years in children born < 28 week gestation in 2005 compared with births in 1991/2 and 1997 [18, 19]. Alongside cognitive impairment, behavioural problems are the most common adverse outcome following EP birth [20]. However, trends over time in behavioural outcomes among EP populations have not been investigated.

In the present study, we compare behavioural outcomes at 11 years of age between children born at 22–25 week gestation in England in 2006 (EPICure2) and in 1995 (EPICure) with contemporaneous term-born peers. We then compare children in the EPICure2 and EPICure cohorts to ascertain whether behavioural outcomes have changed over time following EP birth.

## Methods

### Population

The EPICure2 cohort comprised all births in England in 2006 at 22–26 week gestation. This cohort has undergone follow-up with previous data collections at birth [9] and 3 years of age [13]. At 11 years of age, a geographical sample was identified based on children who received neonatal care in 17 of the 45 neonatal units and networked hospitals in operation in 2006. Of 1041 survivors at discharge from neonatal care, 482 children were invited to participate in the 11-year assessment. The original EPICure cohort comprised all births at 22–25 week gestation in March–December 1995 in all maternity units in Great Britain and Ireland. To compare outcomes over time for the present study, a sub-group of children born at 22–25 week gestation to women residing in England in each cohort was used. These children are referred to as ‘EP children’ throughout.

Term-born (≥ 37 week gestation) children were recruited as a comparison group from classmates of the EP children in mainstream schools, matched for age (± 3 months) and sex where possible using a similar procedure in both cohorts [17, 21]. For home-educated EP children, the child’s parent(s) were asked to identify a term-born child to be invited to participate. Term-born children were not recruited for EP children in special schools.

### Measures

Parents completed the following measures as part of a larger study questionnaire.

#### Strengths and difficulties questionnaire (SDQ)

The SDQ [22] is a 25-item questionnaire for 4–17 years old, completed by parents for this study, with five symptom domains: emotional, conduct, hyperactivity, peer-relationship problems, and prosocial behaviours. Sub-scale scores are calculated by summing the five item scores in each domain (range 0–10). Summing the emotional, conduct, hyperactivity, and peer-relationships sub-scales gives a total difficulties score (range 0–40); higher scores indicate greater difficulties. Clinically significant difficulties were defined using the total difficulties cut-off score (> 16) for abnormal outcomes using test norms.

#### DuPaul ADHD rating scale (ADHD-RS)

Attention-deficit/hyperactivity disorder (ADHD) symptoms were captured using the ADHD-RS version IV [23] in EPICure and version 5 in EPICure2 [24]. Both versions comprise the same eighteen items assessing symptoms over the preceding 6 months. Nine items comprise the inattention scale and nine items comprise the hyperactivity-impulsivity scale (scores range 0–27); higher scores indicate greater symptoms. Clinically significant difficulties were classified using scores above the 90th centile for sex and age from the ADHD-RS v5 [24].

#### Social communication questionnaire (SCQ)

The SCQ (Lifetime Version) [25], completed by parents, was used to screen for symptoms of autism spectrum disorder (ASD). This comprises 40 items assessing reciprocal social interaction, language and communication, and repetitive, restricted or stereotyped behaviour [26]. The questionnaire generates a total SCQ score (range 0–39); higher scores indicate greater symptomatology and higher risk of ASD. A cut-off score of ≥ 15 is validated to identify children at risk for ASD [26] and was used as the cut-off for clinically significant difficulties in this study.

### Data processing and statistical analysis

Data were pseudonymised and stored within University College London (UCL) Data Safe Haven. Statistical analyses were conducted using IBM SPSS Statistics for Windows v25.0 [27].

Demographic data and group characteristics were summarised using descriptive statistics. Dropout analyses were conducted to explore whether EP children assessed at 11 years differed significantly from (1) those not assessed 11 years and (2) those assessed at 2–3 years within each cohort. Inferential statistics were used to assess differences in outcomes between children born EP and at term in their respective cohort, as well as differences between EP children in the EPICure and EPICure2 cohorts. Missing data were prorated at the sub-scale level where there were sufficient items completed. Continuous data were analysed using linear regression models with adjustment for confounders. As the data displayed heteroscedasticity and were not strictly normally distributed, a heteroscedasticity-consistent standard error adjustment was applied to all linear regression models using the Huber–White Sandwich Estimate and the dataset was deemed large enough to overcome the non-normality. Results are reported as differences in mean scores with 95% confidence intervals (CI). Comparisons of rates of clinically significant difficulties were analysed using binary logistic regression models, generating odds ratios (OR) with 95% CI.

Z-scores were calculated for raw scores on each scale using the term-born group in each cohort as reference. Using z-scores for comparison between EP children in EPICure and EPICure2 accounts for population shifts in outcomes over time and allows comparisons of effect sizes between scales with different score ranges.

#### Confounders

Adjustments for potential confounders were made in both linear and binary logistic regression models. These included sex, index of multiple deprivation (IMD) decile at age 11 years, and presence of severe disability for comparisons between EP and term-born children in each cohort. IMD [28] is a widely used measure of relative socio-economic deprivation. Areas in England are ranked from decile 1 (most deprived) to decile 10 (least deprived) based on income, employment, health, education, crime, housing, and environment. Deciles using population data for the time point closest to assessment were used for each cohort. Severe disability was classified as any one of the following: Mental Processing Index (IQ) > 3SD below mean for term-born children (score ≤ 70 for EPICure; ≤ 66 for EPICure2), Gross Motor Function Classification System (GMFCS)/Manual Ability Classification System (MACS) level ≥ 3, no useful hearing with aids, no useful vision, or only sees gross light/movement.

Additional covariates were included in the analyses of outcomes between EP children in the two cohorts: age at assessment, gestational age, birthweight z-score (based on UK population data), multiple births, and maternal age at birth.

## Results

### Study population and drop-out analysis

Of the 482 EP children born at 22–26 week gestation invited to participate in the EPICure2 11-year assessment, 200 children were assessed alongside 143 term-born children. This represented 41.5% of EP children invited and 19.4% of the total EPICure2 cohort alive at 3 years. In the EPICure cohort, 219 EP children born at 22–25 week gestation in Great Britain and Ireland and 153 term-born children were assessed at 11 years of age. Derivation and justification of the EPICure2 and EPICure samples have been described previously [17, 21].

**Fig. 1:**
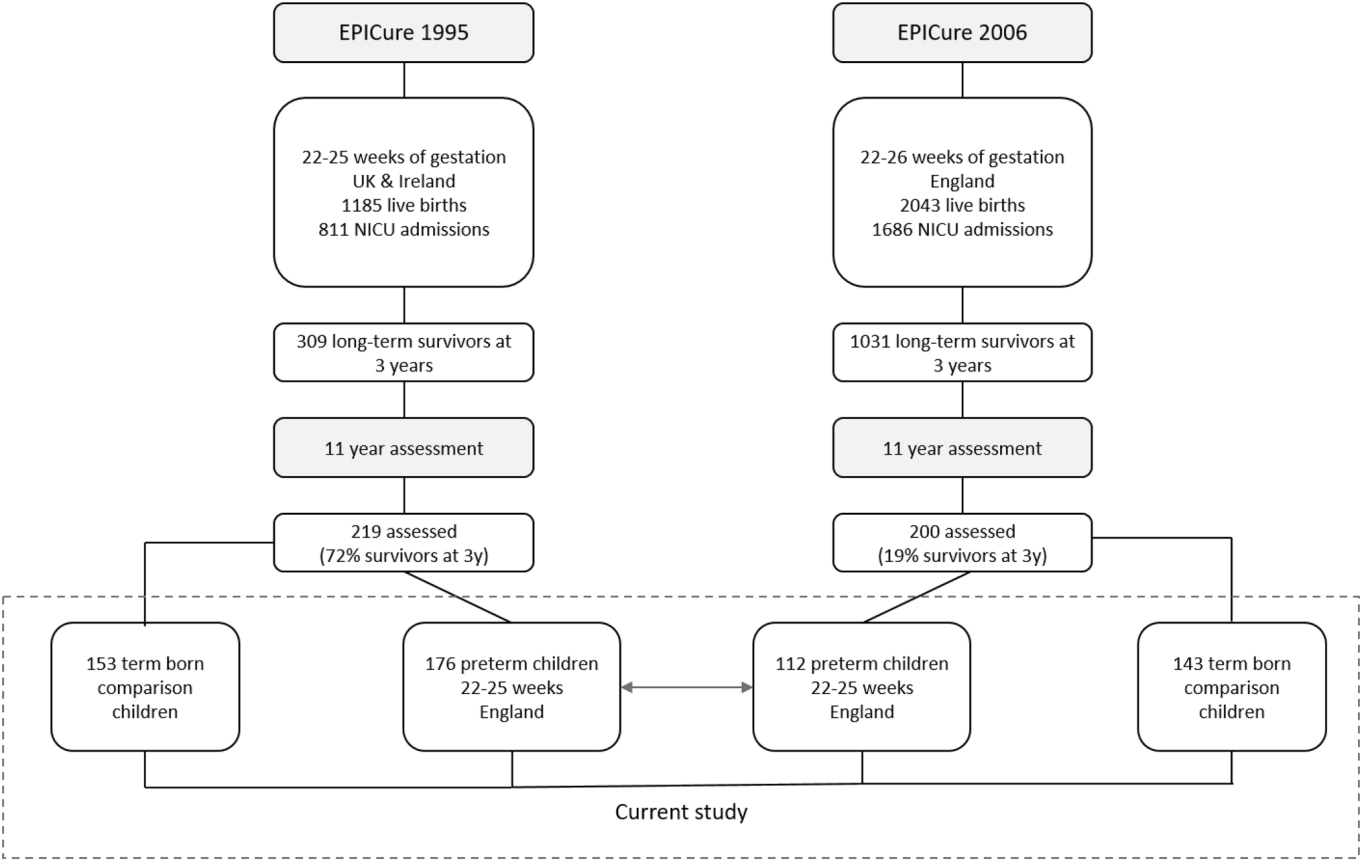
Derivation of the two samples of EP and term-born comparison children assessed at 11 years in the EPICure (1995) and EPICure2 (2006) cohorts

To investigate change in outcomes between the two cohorts, data for a comparable sub-group of EP children born at 22–25 week gestation to mothers resident in England in the EPICure (*n = *176) and EPICure2 cohort (*n = *112) were used (see Fig. 1). For these sub-groups, drop-out analyses revealed similar frequencies of perinatal and maternal characteristics between EP children assessed at 11 years and the whole cohort of EP children alive 2–3 years (Table S1A, Online Resource), as well as those not assessed at 11 years (Table S1B, Online Resource) in both cohorts. There were lower rates of severe disability at 2–3 years in EP children assessed at 11 years in EPICure versus those not assessed [19% vs 31%, χ^2^(1,*N = *235) = 3.809), *p = *0.051].

Children in the EPICure2 cohort were assessed at a higher mean age compared to the EPICure cohort (11.8 years vs. 10.9 years, p < 0.001). The two cohorts had similar distributions of sex, mean IMD decile, and severe disability at 11 years in EP and term-born children (Table 1). The majority of EP children in both cohorts attended mainstream schools (EPICure: 86.4%; EPICure2: 84.8%), one child (0.9%) in EPICure2 was home-educated, and the remaining children attended special schools (13.6% and 14.3%, respectively). All term-born children attended mainstream schools. There was no significant difference in the proportions of EP children who attended mainstream and special schools (χ^2^(1,*N = *287) = 0.034, *p = *0.853) in the EPICure and EPICure2 cohorts. Parent questionnaires were returned more frequently and more parents completed the SDQ for children in EPICure compared to EPICure2. No significant differences in response rates to the ADHD-RS and SCQ were observed between the two cohorts. There were no significant differences in demographic data at 11 years or questionnaire completion rates between EP and term-born children assessed in each of the cohorts (Table 1).

**Table 1:** Characteristics of extremely preterm (born 22–25 week gestation in England) and term-born children in the EPICure and EPICure2 cohorts assessed at 11 years of age

	EPICure 1995 22–25 weeks, born in England			EPICure2 2006 22–25 weeks, born in England			EPICure2 vs. EPICure
	EP [A]	Term [B]	[A vs. B] *p*^a^	EP [C]	Term [D]	[C vs. D] *p*^a^	[C vs. A]*P*^a^
Children assessed (*n*)	176	153	–	112	143	–	–
Age at assessment, mean (SD)	10.9 (0.4)	10.9 (0.5)	0.190	11.9 (0.5)	11.8 (0.6)	0.109	** < 0.001**
Male sex, %^b^ (*n*)	45.5% (80)	41.8% (64)	0.509	50.0% (56)	44.1% (63)	0.345	0.451
IMD decile at 11 years, mean (range)	5.1 (1–10) [*n = *174]	5.7 (1–10) [*n = *123]	0.075	4.9 (1–10) [*n = *111]	5.4 (1–10) [*n = *138]	0.248	0.604
Severe disability, %^b,c^ (*n*)	18.2% (32)	0% (0)	** < 0.001**	25.9% (29)	0% (0)	** < 0.001**	0.083
Parent questionnaire returned, %^b^ (*n*)	94.9% (167)	96.7% (148)	0.408	83.0% (93)	87.4% (125)	0.325	**0.001**
Parent SDQ complete, %^b^ (*n*)	94.9% (167)	96.7% (148)	0.408	87.5% (98)	89.5% (128)	0.616	**0.024**
Parent ADHD-RS* complete, %^b^ (*n*)	85.2% (150)	91.5% (140)	0.079	81.3% (91)	87.4% (125)	0.175	0.373
SCQ complete, %^b^ (*n*)	82.4% (145)	89.5% (137)	0.064	79.5% (89)	83.9% (120)	0.359	0.536

^a^χ^2^ for difference between group proportions and independent samples t test for difference in means

^b^% of those assessed at 11 years

^c^Severe Disability: one or more of the following: Mental Processing Index > 3SD below control mean (< 67), GMFCS/MACS ≥ 3, no useful hearing with aids, no useful vision or only sees gross light/movement

### Dimensional symptom scores

#### EP versus term-born children

EP children had higher mean scores than term-born peers in both the EPICure and EPICure2 cohorts across all measures, indicating greater symptom severity. Differences in mean scores reduced slightly after adjustment for confounders (Table 2).

**Table 2:** Raw scores and z-scores among extremely preterm (born 22–25 week gestation in England) and term-born children in the EPICure and EPICure2 cohorts assessed at 11 years

	EPICure 1995 22–25 weeks		EPICure2 2006 22–25 weeks		EPICure EP vs Term [A vs. B]		EPICure 2 EP vs Term [C vs. D]		EPICure2 vs EPICure (EP) [C vs A]	
	EP [A]	Term [B]	EP [C]	Term [D]						
	Mean (SD)	Mean (SD)	Mean (SD)	Mean (SD)	Unadjusted Δ (95%CI)	Adjusted Δ^a^ (95% CI)	Unadjusted Δ (95%CI)	Adjusted Δ^a^ (95% CI)	Unadjusted Δ (95%CI)	Adjusted Δ^b^ (95% CI)
Strengths and difficulties questionnaire (SDQ)										
	*N = *167	*N = *148	*N = *98	*N = *128						
Total difficulties raw score	11.3 (7.4)	6.2 (6.0)	12.1 (7.2)	5.6 (4.9)	5.1 (3.7, 6.6)**	4.5 (2.9, 6.1)**	6.5 (4.9, 8.2)**	4.8 (3.0, 6.7)**	0.8 (− 1.0, 2.6)	1.0 (− 1.2, 3.2)
Total difficulties z-score^c^	0.9 (1.2)	0 (1)	1.3 (1.5)	0 (1)	0.9 (0.6, 1.1)**	0.7 (0.5, 1.0)**	1.3 (1.0, 1.7)**	1.0 (0.6, 1.4)**	0.5 (0.1, 0.8)*	0.5 (0.1, 0.9)*
Du Paul attention-deficit/hyperactivity rating scale IV/5 (ADHD-RS IV/5)										
	*N = *150	*N = *140	*N = *91	*N = *125						
Inattention sub-scale raw score	8.9 (7.0)	3.4 (4.0)	11.2 (7.7)	3.9 (4.4)	5.6 (4.3, 6.9)**	4.6 (3.2, 6.0)**	7.3 (5.6, 9.1)**	5.8 (3.9, 7.7)**	2.3 (0.3, 4.2)*	1.7 (− 0.7, 4.2)
Inattention z-score^c^	1.4 (1.7)	0 (1)	1.7 (1.8)	0 (1)	1.4 (1.1, 1.7)**	1.1 (0.8, 1.5)**	1.7 (1.3, 2.1)**	1.3 (0.9, 1.7)**	0.3 (− 0.2, 0.7)	0.2 (− 0.4, 0.7)
	*N = *150	*N = *140	*N = *92	*N = *125						
Hyperactivity-impulsivity sub-scale raw score	5.8 (5.8)	2.6 (4.1)	6.0 (6.2)	1.8 (2.4)	3.2 (2.1, 4.4)**	2.4 (1.2, 3.7)**	4.2 (2.9, 5.5)**	3.1 (1.7, 4.5)**	0.2 (− 1.4, 1.8)	0.4 (− 1.7, 2.4)
Hyperactivity-impulsivity z-score^c^	0.8 (1.4)	0 (1)	1.7 (2.6)	0 (1)	0.8 (0.5, 1.1)**	0.6 (0.3, 0.9)**	1.7 (1.2, 2.3)**	1.3 (0.7, 1.9)**	1.0 (0.4, 1.5)*	1.0 (0.3, 1.7)*
Social communication questionnaire (SCQ)										
	*N = *145	*N = *137	*N = *89	*N = *120						
SCQ total raw score	8.0 (7.4)	3.2 (3.4)	10.8 (8.6)	3.1 (3.0)	4.8 (3.5, 6.1)**	3.3 (2.0, 4.6)**	7.7 (5.8, 9.6)**	4.6 (3.0, 6.2)**	2.8 (0.7, 5.0)*	1.3 (− 1.3, 4.0)
SCQ total z-score^c^	1.4 (2.1)	0 (1)	2.5 (2.8)	0 (1)	1.4 (1.0, 1.8)**	1.0 (0.6, 1.3)**	2.5 (1.9, 3.2)**	1.5 (1.0, 2.0)**	1.1 (0.5, 1.8)*	0.7 (− 0.1, 1.5)

Statistically significant results are shown in bold

Δ mean difference

^a^Multiple linear regression models with Huber–White correction for heteroscedasticity–adjusted for sex, IMD at 11 years, and severe disability

^b^Multiple linear regression models (with Huber–White)—adjusted for sex, gestational age, birthweight z-score, IMD at 11y, multiple births, maternal age at birth, age at assessment, and severe disability

^c^z-scores calculated using contemporaneous term-born group scores

*p < 0.05; ** *p* < 0.001

Within each cohort, z-scores were computed using the respective term-born comparison group as the reference. The adjusted difference in mean z-score between EP and term-born children in SDQ total difficulties score was 0.7 (95% CI [0.5, 1.0]) in EPICure and 1.0 (95% CI [0.6, 1.4]) in EPICure2. EP children in both cohorts scored over 1 SD higher than term-born children on the ADHD-RS inattention scale (EPICure: adjusted difference in mean z-score 1.1 [0.8, 1.5], EPICure2: 1.3 [0.9, 1.7]). Smaller adjusted differences in z-scores between EP and term-born children were observed for hyperactivity-impulsivity in EPICure (0.6, 95%CI [0.3, 0.9]) than in EPICure2 (1.3, [0.7, 1.9]), but both were statistically significant. Adjusted mean SCQ total z-scores of 1.0 (95% CI [0.6, 1.3]) and 1.5 [1.0, 2.0] were observed for EP children in the EPICure and EPICure2 cohorts, respectively.

#### EP children in EPICure2 (2006) versus EPICure (1995)

**Fig. 2:**
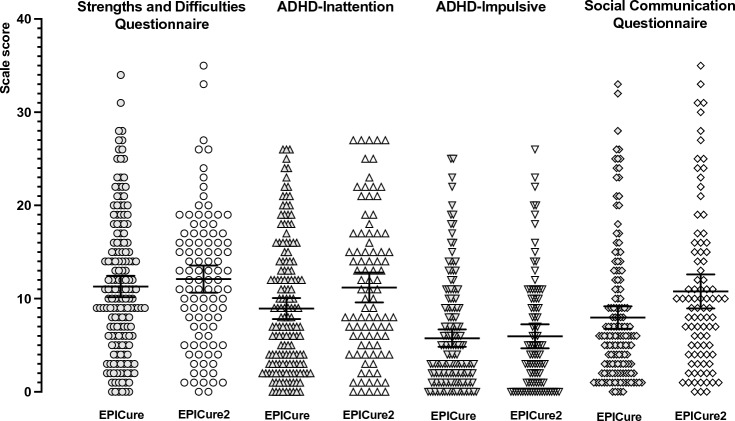
Distribution of SDQ total difficulties, ADHD-RS inattention, ADHD-RS hyperactivity-impulsivity, and SCQ total scores for EP children in EPICure (1995) and EPICure2 (2006). Bars indicate group mean and 95% confidence interval

Comparing EP children in the two cohorts using raw scores, no significant differences were observed for any measure after adjustment for confounders (Table 2, Fig. 2). However, comparing outcomes between EP children in the two cohorts using z-scores, EP children in EPICure2 had significantly higher unadjusted z-scores compared with EP children in EPICure on all measures, apart from the ADHD-RS inattention scale (Table 2). After adjustment for confounders, differences in mean z-scores remained significant for SDQ total difficulties and ADHD hyperactivity-impulsivity. There were no significant differences between raw scores for term-born children in the two cohorts in any of the measures after adjustment for sex, IMD at 11 years, age at assessment, and severe disability (Table S2, Online Resource).

### Clinically significant difficulties

#### EP versus term-born children

**Fig. 3:**
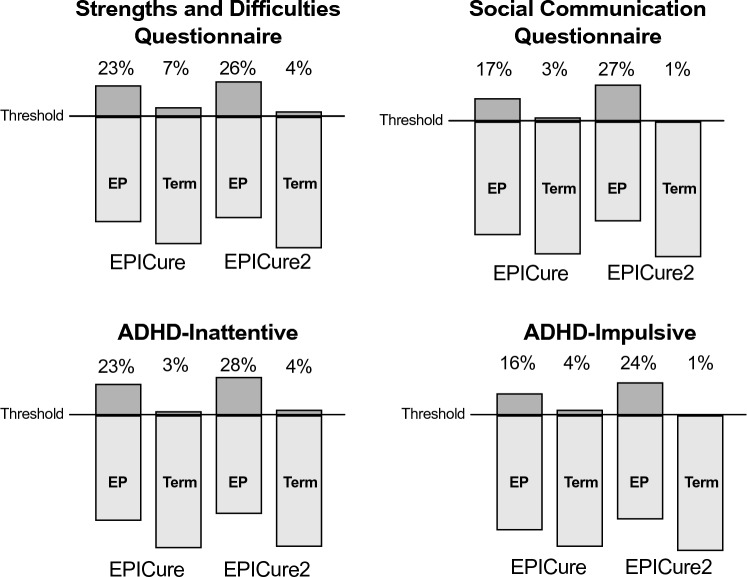
Proportions (%) of EP and term-born children in EPICure (1995) and EPICure2 (2006) scoring above cut-offs for clinically significant difficulties

The proportion of EP and term-born children scoring above the cut-off for clinically significant difficulties on each measure is shown in Fig. 3. Compared to term-born children, greater proportions of EP children scored above the cut-off for clinically significant difficulties on all measures in both cohorts, except for EP versus term-born children in EPICure for the SCQ (Table 3).

**Table 3:** Prevalence of clinically significant difficulties among EP (born 22–25 week gestation in England) and term-born children in the EPICure and EPICure2 cohorts at 11 years of age

	EPICure 1995 22–25 weeks			EPICure2 2006 22–25 weeks		EPICure EP vs Term [A vs. B]		EPICure 2 EP vs Term [C vs. D]		EPICure2 vs EPICure (EP) [C vs A]	
	EP [A]		Term [B]	EP [C]	Term [D]						
	% (n)	% (n)		% (n)	% (n)	Unadjusted OR (95%CI)	Adjusted OR^a^ (95% CI)	Unadjusted OR (95%CI)	Adjusted OR^a^ (95% CI)	Unadjusted OR (95%CI)	Adjusted OR^b^ (95% CI)
Strengths and difficulties questionnaire											
	*N = *167	*N = *148		*N = *98	*N = *128						
SDQ total difficulties (score > 16)	23.4% (39)	6.8% (10)		25.5% (25)	3.9% (5)	4.2 (2.0, 8.8)**	3.3 (1.5, 7.3)*	8.4 (3.1, 23.0)**	4.7 (1.6, 14.0)*	1.1 (0.6, 2.0)	1.6 (0.7, 3.6)
Du Paul ADHD-RS IV/5											
	*N = *150	*N = *140		*N = *91	*N = *125						
Inattention score > 90th centile^c^	22.7% (34)	2.9% (4)		27.5% (25)	4.0% (5)	10.0 (3.4, 28.9)**	6.8 (2.3, 20.1)*	9.1 (3.3, 24.9)**	7.6 (2.7, 21.8)**	1.3 (0.7, 2.4)	1.9 (0.8, 4.6)
	*N = *150	*N = *140		*N = *92	*N = *125						
Hyperactivity-impulsivity > 90th centile^c^	16.0% (24)	4.3% (6)		23.9% (22)	0.8% (1)	4.3 (1.7, 10.8)*	3.5 (1.2, 9.7)*	39.0 (5.1, 295.3)**	26.5 (3.3, 210.1)*	1.7 (0.9, 3.2)	1.7 (0.6, 4.3)
Social communication questionnaire											
	*N = *145	*N = *137		*N = *89	*N = *120						
ASD/autistic disorder (SCQ score ≥ 15)	16.6% (24)	2.9% (4)		27.0% (24)	0.8% (1)	6.6 (2.2, 19.6)*	3.0 (0.9, 9.6)	43.9 (5.8, 332.2)**	20.7 (2.5, 167.9)*	1.9 (1.0, 3.5)	2.1 (0.7, 6.6)

Statistically significant results are shown in bold

^a^Binary logistic regression models–adjusted for sex, IMD at 11 years and severe disability

^b^Binary logistic regression–adjusted for sex, gestational age, birthweight Z score, IMD at 11y, multiple births, maternal age at birth, age at assessment and severe disability

^c^90th centile score specific for sex and age at assessment, using ADHD-RS 5 for both cohorts

* *p* < 0.05; ** *p* < 0.001

Adjusted ORs for clinically significant difficulties among EP compared with term-born children ranged from 3.0 to 6.8 in EPICure and 4.7 to 26.5 in EPICure2 (Table 3). Only a very small percentage of term-born children scored above the cut-off for clinically significant difficulties for the ADHD-RS hyperactivity-impulsivity scale and the SCQ in EPICure2, which resulted in large confidence intervals. Results should therefore be interpreted with caution.

#### EP children in EPICure2 versus EPICure

**Fig. 4:**
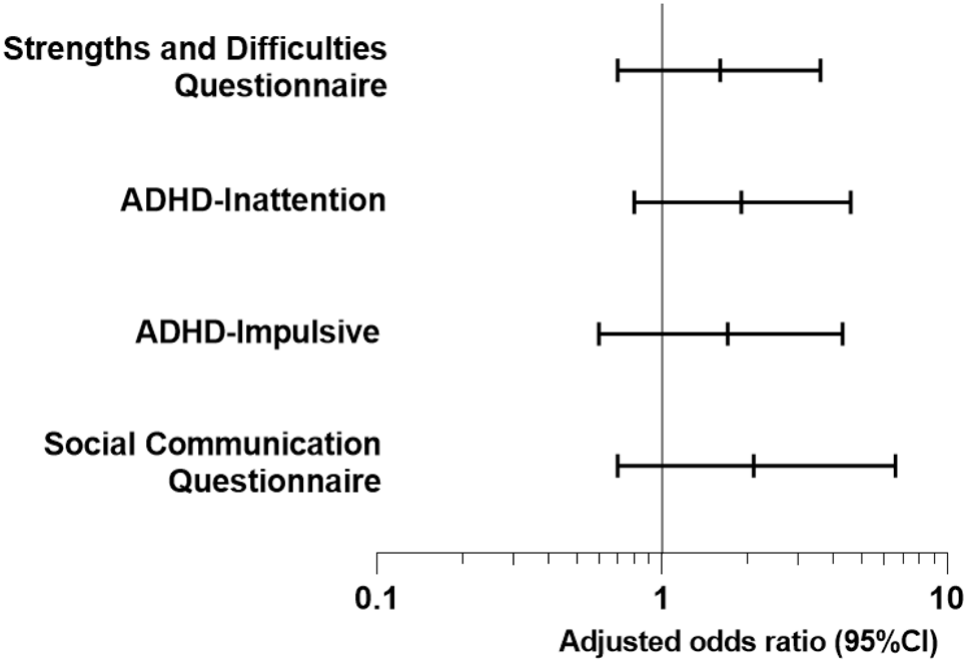
Adjusted Odds Ratios for risk of clinically significant difficulties in extremely preterm born children in EPICure2 (2006) vs EPICure (1995)

No differences were found in the proportion of EP children scoring above the threshold for clinically significant difficulties between the two cohorts across all measures (Fig. 4, Table 3). Lower proportions of term-born children in EPICure2 had clinically significant difficulties for all measures except the ADHD-RS inattention scale, relative to term-born children in EPICure. Adjusted odds ratios for term-born children in EPICure2 compared with those in EPICure were all less than 1, indicating that term-born children in EPICure2 had lower odds of clinically significant difficulties than those in EPICure, although only the hyperactivity-impulsivity score was statistically significant (Table S3, Online Resource).

## Discussion

In this prospective population-based multi-cohort study, children born EP at 22–25 weeks of gestation in England consistently had greater parent-reported behavioural, attention, hyperactivity-impulsivity, and social-communication difficulties than term-born children. Comparing outcomes of EP children in the two cohorts born 11 years apart (EPICure: 1995, EPICure2: 2006), we found no significant improvement over time in mean scores or in the proportion of EP children with clinically significant difficulties across all measures. Additionally, relative to their contemporaneous term-born peers, EP children in EPICure2 had higher z-scores than EP children in EPICure for SDQ total difficulties and the ADHD-RS hyperactivity-impulsivity scale. This is likely to be the result of a combination of slightly higher raw scores for EP children and lower raw scores for term-born children in EPICure2 compared with EPICure. This suggests a widening of the gap in behavioural outcomes for EP children relative to their term-born peers over time.

The persistent excess of behavioural difficulties for children born EP compared to term-born children in this study is in keeping with the existing research across varying ages throughout childhood [4, 29–35]. The lack of improvement in behavioural outcomes over time in this study is in accordance with findings of no change in neurodevelopmental, cognitive, or educational outcomes in the same children [17] and in studies of outcomes in other consecutive cohorts during childhood [14, 16]. Furthermore, the worsening of some behavioural outcomes for EP children in EPICure2 relative to term-born peers is similar to the VICS cohorts, which demonstrated worsening executive function [18], academic achievement [16], and motor outcomes over time [19]. Currently, there are no other studies examining changes over time in behavioural outcomes between cohorts of EP children; however, our findings are in keeping with the meta-analysis by Mathewson et al. [4] which showed no effect of year of birth (before 1990 vs 1990 or later) on rates of most mental health outcomes investigated.

The worsening of behavioural outcomes for EP children born in 2006 compared with those born in 1995 could be explained by increased survival rates for the later cohort. If babies who previously would have died during the neonatal period, who were therefore sicker and potentially of more immature gestational ages, survived, it might be expected that these children would have worse outcomes than more mature infants. Survival at 23 weeks of gestation was 9.5% higher in EPICure2 compared to EPICure, although rates of major neonatal morbidity did not differ between cohorts [9]. However, ex-utero brain growth and development could still be affected to a greater extent for infants of more immature gestational ages leading to increased risks of behavioural difficulties. Adjusting for severe disability and gestational age during regression analysis will have reduced the impact of these differences, although not removed them entirely.

On the other hand, secular trends in mental health in the general population suggest that emotional problems and conduct disorders may be increasing [36] and there has also been a significant increase in the rates of ASD [37, 38]. Rates of ADHD, however, have been more stable [36]. However, the trend for increased mental health problems was not observed for term-born children in this study. No significant differences in dimensional scores were observed between term-born children in EPICure and EPICure2 after adjustment for confounders. Furthermore, lower proportions of term-born children in EPICure2 had clinically significant difficulties for all measures except inattention, relative to term-born children in EPICure. The widening of the gap between EP and term-born children observed in EPICure2 is therefore concerning and may imply that there has been an even greater trend towards increased difficulties in EP children than the general population.

There is also the potential for cultural shifts in the way parents completed the measures between the two cohorts. The analysis of z-scores accounts for cultural shifts in the general population; however, there could be a greater shift among parents of EP children relative to term-born children. Increasing awareness of the types of difficulties children born EP may experience could lead parents to recognise and rate their EP children with more difficulties than previously. This could mask any subtle improvements in outcomes over time and result in greater between-group differences in outcomes for children in the EPICure2 cohort compared with those in EPICure. However, the lack of improvement in more objectively measured neurodevelopmental and cognitive outcomes between EPICure and EPICure2 [17] assessed by trained medical professionals and psychologists blinded to group may render this explanation less likely.

### Strengths and limitations

The strengths of this study are the prospective longitudinal follow-up of EP children born across two population-based cohorts 11 years apart, the recruitment of term-born comparison children for each cohort to account for secular trends in outcomes, and the use of well-established and validated dimensional measures of behavioural outcomes. Recruiting term-born children from the same mainstream schools as EP participants reduced differences in educational experiences and socio-economic status between groups. However, the term-born children in both cohorts had lower SDQ scores than might be anticipated, with 6.8% in EPICure and 3.9% in EPICure2 scoring above the cut-off for clinically significant difficulties based on scores > 90th centile of the general population. Similarly, less than 5% of term-born children scored > 90th centile on the ADHD-RS inattention and hyperactivity-impulsivity scales, and only 0.8% of term-born children in EPICure2 scored > 90th centile score for the hyperactivity-impulsivity scale. Consequently, the term-born comparison children may represent a ‘healthier’ group than the general population in terms of behavioural outcomes. This may have inflated the odds of behavioural difficulties in EP children and widened the confidence intervals. A possible reason for this is that recruiting term-born comparison children from only mainstream schools resulted in a term-born group which have less behavioural difficulties than the general population.

A further strength is the consistent use of the same measures across the two cohorts. However, the 5^th^ edition of the DuPaul ADHD-RS [24] was published between the 11 year assessment of the EPICure and EPICure2 cohorts, making the 4th edition [23] unavailable for use in the EPICure2 Study. The 18 items included in the measure and the scoring of items are identical in both editions; however, the order of items does differ. It is not clear what effect this has, but we believe that the 4th and 5th editions are sufficiently comparable to evaluate the change over time robustly. We have applied the ADHD-RS 5th edition 90th centile normative cut-offs to both cohorts for consistency. The same SDQ and SCQ editions were used for both cohorts.

To our knowledge, this is the first study investigating behavioural outcomes among EP children across two prospective national population-based cohorts. Participant attrition is often a challenge in prospective longitudinal studies. A limitation of this study is the lower response rates in EPICure2 than EPICure. Consequently, the relatively small sample size precludes conducting sub-group analyses. Children of lower socio-economic status, male sex, born to multiparous or younger mothers, and with more severe disability are more likely to drop out of follow-up [39], biasing results. However, our drop-out analysis revealed satisfactory matching of baseline perinatal, maternal demographic, and socio-economic characteristics between those assessed at 11 years compared with those not assessed, and with those assessed at 2–3 years within each cohort. Children assessed at 11 years had lower rates of severe disability at 2–3 years than those not assessed at 11 years in the EPICure cohort (19.3% vs 31.3%). Behavioural outcomes in the EPICure cohort assessed at 11 years may therefore underestimate the prevalence in the whole cohort, which could have contributed to the worsening of outcomes for the EPICure2 cohort. However, adjustments for confounders were applied to all regression analyses to minimise bias from cohort differences. A further limitation is that our results are based on parent-report measures and not diagnostic data. However, the SDQ has been shown to have good diagnostic accuracy for identifying EP children with the diagnoses of psychiatric disorders made using the Development and Well-being Assessment [40]. Further research into the change over time in psychiatric diagnoses of EP children is needed and will be the subject of future analyses in these cohorts.

## Conclusions

Despite improvements in neonatal care and increased survival, children born EP continue to have more behavioural difficulties than their term-born peers. No improvements in behavioural, attention, and social-emotional outcomes were observed between EP children born 2006 compared with those born in 1995. Additionally, EP children in EPICure2 appear to have worse outcomes relative to their term-born peers in some areas than EP children in EPICure. Continued research into cohorts of EP children born more recently is important to determine if improvements in survival and neonatal care since 2006 will in time lead to improvements in long-term outcomes. This should be coupled with further research into potential interventions to support EP children who have attention, social and emotional difficulties, and their families.

### Supplementary Information

Below is the link to the electronic supplementary material.

Supplementary file1 (DOCX 29 KB)

## Data Availability

Data are available subject to the EPICure Data Sharing Policy (www.epicure.ac.uk) and will be available as part of the RECAP preterm Cohort Platform (https://recap-preterm.eu).
