# Supplementary material for: Comparing behavioural outcomes in children born extremely preterm between 2006 and 1995: the EPICure studies
Source: Eur Child Adolesc Psychiatry. 2023 Jul 11;33(5):1517–28. doi: 10.1007/s00787-023-02258-w (PMC11098736; doi:10.1007/s00787-023-02258-w)
Supplement: Supplementary file 1 — Supplementary file1 (DOCX 29 KB) [file 787_2023_2258_MOESM1_ESM.docx]

**Supplementary Material**

**Article title:** Comparing behavioural outcomes in children born extremely preterm between 2006 and 1995: the EPICure studies

**Journal:** European Child and Adolescent Psychiatry

**Authors:** Jennifer Larsen, Puja Kochhar, Dieter Wolke, Elizabeth S. Draper, Neil Marlow, Samantha Johnson

**Corresponding Author:** Professor Samantha Johnson, University of Leicester (Department of Population Health Sciences), George Davies Centre, University Road, Leicester, LE1 7RH, United Kingdom. sjj19@leicester.ac.uk

**Table S1:** Drop-out analysis: Characteristics of extremely preterm children born at 22-25 week gestation in England in the EPICure and EPICure2 cohorts who were assessed at 11 years versus (A) the whole sample and (B) those not assessed at 11 years.

| A) | EPICure 1995^a^ | | | EPICure2 2006^a^ | |
| --- | --- | --- | --- | --- | --- |
|  | Assessed sample at 2.5y | Assessed sample at 11 years | Assessed sample at 3y | | Assessed sample at 11 years |
|  | n=260 | n=176 | n=584 | | n=112 |
| Gestational age, %(n/N) |  |  |  | |  |
| 25w | 59% (153/260) | 56% (99/176) | 58% (341/584) | | 62% (69/112) |
| 24w | 32% (83/260) | 33% (58/176) | 30% (177/584) | | 25% (28/112) |
| ≤23w | 9% (24/260) | 11% (19/176) | 11% (66/584) | | 13% (15/112) |
| Birthweight (g), mean (range) | 748 (480–1040) | 746 (480–1040) | 735 (449–1125) | | 740 (479–1059) |
| Birthweight z scores, mean (SD) | -0.2 (0.8)  [n=259] | -0.1 (0.8)  [n=175] | -0.2 (07)  [n=581] | | -0.2 (0.7) |
| Male sex, %(n/N) | 49% (127/260) | 46% (80/176) | 48% (280/584) | | 50% (56/112) |
| Multiple birth, %(n/N) | 26% (67/260) | 29% (51/176) | 22% (129/584) | | 24% (27/112) |
| Maternal age, mean (range) | 28.5 (14–43)  [n=259] | 28.8 (14–43)  [n=175] | 29.4 (15–54) | | 30.8 (16–54) |
| IMD decile |  |  |  | |  |
| at birth, mean (range) | -- | -- | 4.2 (1–10)  [n=581] | | 4.3 (1–10)  [n=111] |
| at 11y, mean (range) | -- | 5.1 (1–10)  [n=174] | -- | | 4.9 (1–10)  [n=111] |
| Severe Disability at 2-3y^b^, %(n/N) | 23% (53/235) | 19% (33/171) | 16% (53/325) | | 15% (13/88) |
|  | | | | | |
| B) | **EPICure 1995^a^** | | **EPICure2 2006 ^a^** | | |
|  | Not assessed at 11years | Assessed sample at 11 years | Not assessed at 11 y | | Assessed sample at 11 years |
|  | n=84 | n=176 | n=472 | | n=112 |
| Gestational age, %(n/N) |  |  |  | |  |
| 25w | 64% (54/84) | 56% (99/176) | 58% (272/472) | | 62% (69/112) |
| 24w | 30% (25/84) | 33% (58/176) | 32% (149/472) | | 25% (28/112) |
| ≤23w | 6% (5/84) | 11% (19/176) | 11% (51/472) | | 13% (15/112) |
| Birthweight (g), mean(range) | 753 (530-997) | 746 (480–1040) | 734 (449-1125) | | 740 (479–1059) |
| Birthweight z scores, mean(SD) | -0.2 (0.7) | -0.1 (0.8)  [n=175] | -0.3 (0.7)  [n=469] | | -0.2 (0.7) |
| Male sex, %(n/N) | 56% (47/84) | 46% (80/176) | 48% (224/472) | | 50% (56/112) |
| Multiple birth, %(n/N) | 19% (16/84) | 29% (51/176) | 22% (102/472) | | 24% (27/112) |
| Maternal age, mean(range) | 27.8 (16-43) | 28.8 (14–43)  [n=175] | 29.0 (15-51) | | 30.8 (16–54) |
| IMD decile |  |  |  | |  |
| at birth, mean(range) | -- | -- | 4.2 (1-10)  [n=470] | | 4.3 (1–10)  [n=111] |
| at 11y, mean(range) | -- | 5.1 (1–10)  [n=174] | -- | | 4.9 (1–10)  [n=111] |
| Severe Disability at 2-3y^b^ | 31% (20/64) | 19% (33/171) | 17% (40/237) | | 15% (13/88) |
|  |  |  |  | |  |

^a^ Sub-group of EPICure and EPICure2 cohorts born 22-25 weeks of gestation, in England
^b^ Severe disability: developmental quotient <3SD below mean for age, GMFCS ≥3, blindness or profound sensorineural hearing loss not improved by aids

**Table S2** Comparison of raw scores and z-scores among EPICure and EPICure2 term-born children at 11 years

|  | EPICure1 1995  22-25 weeks | | EPICure2 2006  22-25 weeks | | EPICure2 vs 1 TERM-BORN  [D vs B] | |
| --- | --- | --- | --- | --- | --- | --- |
|  | EP  [A] | Term  [B] | EP  [C] | Term  [D] |  |  |
|  | Mean (SD) | Mean (SD) | Mean (SD) | Mean (SD) | Unadjusted Δ (95%CI) | Adjusted Δ ^a^  (95% CI) |
| Strengths and Difficulties Questionnaire | | | | | | |
|  | n=167 | n=148 | n=98 | n=128 |  |  |
| Total difficulties raw score | 11.3 (7.4) | 6.2  (6.0) | 12.1 (7.2) | 5.6 (4.9) | -0.6  (-1.9, 0.7) | -0.7  (-2.2, 0.9) |
| Total difficulties Z-score ^b^ | 0.9  (1.2) | 0  (1) | 1.3 (1.5) | 0  (1) | --- | --- |
| Du Paul ADHD-RS IV/5 | | | | | | |
|  | n=150 | n=140 | n=91 | n=125 |  |  |
| Inattention sub-scale raw score | 8.9  (7.0) | 3.4 (4.0) | 11.2  (7.7) | 3.9  (4.4) | 0.5  (-0.5, 1.5) | -0.3  (-1.7, 1.0) |
| Inattention Z-score ^b^ | 1.4  (1.7) | 0  (1) | 1.7  (1.8) | 0  (1) | --- | --- |
|  | n=150 | n=140 | n=92 | n=125 |  |  |
| Hyperactivity-impulsivity sub-scale raw score | 5.8  (5.8) | 2.6  (4.1) | 6.0  (6.2) | 1.8  (2.4) | **-0.8**  **(-1.6, 0.001)*** | -0.8  (-2.2, 0.5) |
| Hyperactivity-impulsivity Z-score ^b^ | 0.8  (1.4) | 0  (1) | 1.7  (2.6) | 0  (1) | --- | --- |
| Social Communication Questionnaire | | | | | | |
|  | n=145 | n=137 | n=89 | n=120 |  |  |
| Total SCQ raw score | 8.0  (7.4) | 3.2  (3.4) | 10.8  (8.6) | 3.1  (3.0) | -0.1  (-0.9, 0.7) | -0.1  (-1.0, 0.8) |
| Total SCQ Z-score ^b^ | 1.4  (2.1) | 0  (1) | 2.5  (2.8) | 0  (1) | --- | --- |

Δ mean difference
^a^ Multiple linear regression models with Huber-White corrected for heteroscedasticity - adjusted for sex, IMD at 11y, age at assessment and severe disability
^b^ Z scores calculated using cohort control group scores
* *p*≤0.05

**Table S3** Comparison of prevalence of clinically significant difficulties among EPICure and EPICure2 term-born children at 11 years

|  | EPICure1 1995  22-25 weeks | | EPICure2 2006  22-25 weeks | | EPICure2 vs 1 Term  [d vs b] | |
| --- | --- | --- | --- | --- | --- | --- |
|  | EP  [A] | Term [B] | EP  [C] | Term [D] |  |  |
|  | % (n) | % (n) | % (n) | % (n) | Unadjusted OR  (95%CI) | Adjusted OR ^a^  (95% CI) |
| Strengths and Difficulties Questionnaire | | | | | | |
|  | n=167 | n=148 | n=98 | n=128 |  |  |
| Abnormal SDQ total difficulties (>16) | 23.4%  (39) | 6.8%  (10) | 25.5%  (25) | 3.9%  (5) | 0.6  (0.2, 1.7) | 0.5  (0.1, 2.0) |
| Du Paul ADHD-RS IV/5 | | | | | | |
|  | n=150 | n=140 | n=91 | n=125 |  |  |
| Inattention score >normative 90^th^ centile ^b^ | 26.0%  (39) | 2.9%  (4) | 27.5%  (25) | 4.0%  (5) | 1.4  (0.4, 5.4) | 0.5  (0.1, 2.2) |
|  | n=150 | n=140 | n=92 | n=125 |  |  |
| Hyperactivity-impulsivity >normative 90^th^ centile ^b^ | 18.0%  (27) | 4.3%  (6) | 23.9%  (22) | 0.8%  (1) | 0.2  (0.02, 1.5) | **0.06**  **(0.005, 0.7)*** |
| Social Communication Questionnaire | | | | | | |
|  | n=145 | n=137 | n=89 | n=120 |  |  |
| ASD/autistic disorder (SCQ score ≥15) | 16.6%  (24) | 2.9%  (4) | 27.0%  (24) | 0.8%  (1) | 0.3  (0.03, 2.5) | 0.2  (0.02, 2.8) |

^a^ Binary logistic regression models - adjusted for sex, IMD at 11y, age at assessment and severe disability

^b^ Normative 90^th^ centile score specific for sex and age at assessment, using ADHD-RS 5 for both cohorts

* *p*≤0.05
